# Application of a Mixture of Fly Ash and Solid Waste from Gas Treatment from Municipal Solid Waste Incineration in Cement Mortar

**DOI:** 10.3390/ma18030481

**Published:** 2025-01-21

**Authors:** Alina Pietrzak, Malgorzata Ulewicz, Ewa Kozień, Jacek Pietraszek

**Affiliations:** 1 Faculty of Civil Engineering, Czestochowa University of Technology, Dabrowskiego 69 Street, 42-201 Czestochowa, Poland; alina.pietrzak@pcz.pl (A.P.); malgorzata.ulewicz@pcz.pl (M.U.); 2 College of Management and Quality Sciences, Krakow University of Economics, Rakowicka 27, 31-510 Kraków, Poland; koziene@uek.krakow.pl; 3 Faculty of Mechanical Engineering, Cracow University of Technology, Al. Jana Pawla II 37, 31-864 Kraków, Poland

**Keywords:** cement mortar, fly ash, solid waste from gas treatment, municipal solid waste, civil engineering, mechanical engineering, circular economy

## Abstract

This paper analyzes the effective use of a mixture of fly ash (MSWI-FA) and solid waste from flue gas treatment (MSWI-SW), which are by-products of the municipal waste incineration process. MSWI-FA (19 01 13*) and MSWI-SW (19 01 07*) are classified as hazardous waste due to their toxic metal content and leaching potential, and currently lack practical applications, unlike slag and bottom ash (19 01 12). This study tested these wastes as partial substitutes for natural sand within a range of 0–20% of cement mass. Statistical analysis of the experimental results allowed the creation of good quality models predicting the effect of substitution additives on compressive strength and flexural strength (correlation 0.91 and 0.93, respectively). The mixture with the highest share of substitution additives (40% = 20% + 20%) was characterized by a decrease in compressive strength by 1.3% and flexural strength by 25.8%. Cement mortars synthesized with the waste mixture (up to 20% of each component) showed slightly lower consistency and water absorption than the control mortars. After the frost resistance tests (25 cycles), the flexural and compressive strength showed ambiguous behavior, showing both increases and decreases, indicating that the percentage of waste components alone is an insufficient set of factors for predicting these strength properties. The concentration of metal ions, i.e., Zn, Cu, Pb, Ni, Cu, and Cr, in the eluate after the leaching tests did not exceed the legal levels of pollutants introduced into waters, with the exception of barium. However, its content did not exceed the permissible levels required for waste intended for landfill. Using the mixing plant for this waste in the amount of 20% each, we save about EUR 10 in the cost of purchasing sand (which is 13% of the production costs of 1 m^3^) and EUR 8 in the cost of environmental fees when producing 1 m^3^ of mortar. The proposed technology is compatible with the objectives of a sustainable economy.

## 1. Introduction

**Figure 1:**
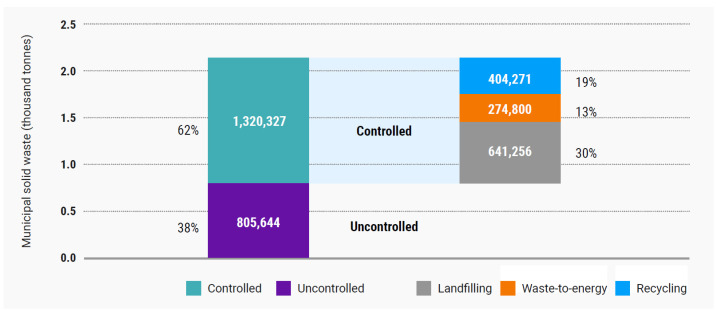
Global destinations of MSW in 2020: controlled (landfilling, waste-to-energy recycling) and uncontrolled [3].

The systematic increase in municipal solid waste (MSW) presents a serious challenge to the natural environment. Accurate forecasting of MSW generation is essential, not only for effective planning but also for optimizing municipal waste management systems. Artificial intelligence systems increasingly manage these systems [1], especially predictive machine learning modeling [2]. According to a report by the International Solid Waste Association [3], in 2023, global MSW production reached 2.1 billion tons. It is estimated that by 2050, MSW generation will rise by 56%, reaching 3.8 billion tons [4]. Municipal solid waste is currently managed in uncontrolled and controlled ways (Figure 1). Of the total municipal waste managed in a controlled manner worldwide, only 13% was thermally treated for energy recovery in 2020. This percentage is somewhat higher in EU countries, where in 2022, out of 229 million tons of MSW generated, 59 million tons (25.8%) were thermally processed [4]. The volume of thermally processed MSW is expected to rise rapidly, as this method is currently regarded as the most efficient technology for MSW management. Thermal processing can reduce MSW mass by approximately 70% and volume by about 90% [5,6] while also generating a significant amount of energy (19%).

As the thermal processing of MSW increases, so will the volume of by-products, which will require safe processing or disposal to protect the environment. From a circular economy perspective, it is crucial that all types of waste, including by-products from MSW thermal processing (e.g., slag, bottom ash, boiler dust, fly ash, and solid residues from flue gas cleaning), are effectively managed [7,8]. This approach is fundamental to sustainable development and minimizing environmental impact. Ingaldi and Ulewicz [9] emphasize the importance of circular economy business models in driving innovation and improving industrial processes, which can be directly applied to waste management in the MSW incineration process.

The thermal conversion of municipal solid waste (MSW) in power plants produces various waste by-products, which are classified under subgroup 19 01 (Table 1) according to the EU Commission Directive [10]. These by-products are categorized as either non-hazardous or hazardous. Certain materials, such as solid waste, aqueous liquid waste from gas treatment, and spent activated carbon, are classified as hazardous. Other wastes, such as fly ash, boiler dust, bottom ash, and slag, may fall into different classifications depending on the type and concentration of pollutants they contain. Accurate pollutant concentration assessment, classification, and assignment of appropriate codes significantly influence waste management practices, including transport, processing, disposal, and recovery.

The waste from the MSW thermal conversion process accounts for about 25–30% of the initial material mass [11,12]. During the thermal processing of municipal solid waste, approximately 300 kg of slag, 5 kg of fly ash, 5 kg of bottom ash, 20 kg of solid residues from flue gas purification, and 12 kg of wet by-products from gas purification are generated [13]. Bottom ash, collected from the bottom of combustion chambers, includes glassy silicate slag, metal objects, glass fragments, ceramics, and minerals (primarily magnetite, quartz, and calcite). In contrast, fly ash consists of fine particles, mostly amorphous, with sulfate and chloride minerals. MSWI fly ash also contains toxic organic compounds (such as polycyclic aromatic hydrocarbons, polychlorinated biphenyls, polychlorinated dibenzodioxins, and furans) as well as heavy metal ions (e.g., Pb, Cr, Zn, Cd, Cu). The main mineral phases in APC residues include halite, sylvite, calcite, anhydrite, quartz, gehlenite, hematite, portlandite, and calcium chloride, and they contain toxic metal ions (e.g., Pb, Cd, Cr).

Fly ash and lime residues from APC, classified as hazardous, are disposed of in specialized landfills (USEPA, 2012; European Commission, 2015) [14,15]. However, landfilling these wastes is costly and results in the loss of materials that could otherwise be utilized in other sectors. For bottom ash, which typically has lower concentrations of toxic metals (such as Zn, Cr, Pb, Cu, Ni, and Cd) compared to fly ash and APC residues, stabilization and solidification processes (using appropriate additives and hydraulic binders) are often applied to convert the waste from hazardous to non-hazardous or neutral. Stabilized and solidified waste can be used in road construction (e.g., in Italy, France, and China), landfill construction (Norway), and clinker production (Japan) [12,16]. Despite these applications, there is still a need for new, efficient, and safe methods for reusing MSWI waste, including hazardous materials. In Poland, MSWI waste undergoes stabilization and solidification before being placed in non-hazardous and neutral waste landfills. This practice is inconsistent with the principles of a circular economy and leads to the loss of valuable materials. Therefore, developing alternative methods for managing these materials, which can provide a viable alternative to landfilling, is essential.

Numerous studies in the literature discuss the use of waste from the thermal processing of municipal solid waste, including fly ash (MSWI-FA), bottom ash (MSWI-BA), and slag (MSWI slag), in the construction sector [15,16,17]. Research has demonstrated that ground bottom ash (MSWI-BA) can serve as a pozzolanic additive to cement [18,19]. Bottom ash has also been utilized as a substitute for natural aggregates in road bases [20] and as an additive in concrete [18,21,22,23,24] and mortars [25,26]. Also, MSWI-FA has been used to create building tiles [27] and geopolymers [28,29], and as an additive to cement [30,31,32,33]. Furthermore, MSWI-FA has been incorporated into slag–cement mortars [30,34] after being melted in an electric furnace at 1450 °C for one hour and subsequently milled. Using MSWI-FA in slag–cement mortar as a partial replacement for Portland cement (10–40%) combined with CaCO_3_ (10–40% by weight) produces composites with high compressive strength after 90 days of curing. Moreover, fly ash MSWI-FA has been used in concrete [18,35] and mortar production [31,36,37,38,39,40]. The use of MSWI-FA causes a decrease in the compressive strength of mortars produced with this waste compared to the control mortars. However, an increase in the compressive strength in concretes containing MSWI waste can be achieved by introducing 0.1% polypropylene fibers [41].

In laboratory tests, a mixture of fly ash and bottom ash (FA + BA) from MSWI incinerators was also used to produce concrete [42,43] and mortar [44,45]. Cement mortars containing a mixture of fly ash and bottom ash (particle size 0.075–4.75 mm), used as a substitute for sand (at levels of 0–40%) and cement (at levels of 0–30%), exhibited higher compressive strength than control mortars [45]. Cement mortars containing a mixture of fly ash and bottom ash (particle size 0.075–4.75 mm), used as a substitute for sand (0–40%) and cement (0–30%), demonstrated higher compressive strength than the control mortars [45]. MSWI slag has also been used as a substitute for natural aggregates in concretes [46,47] and mortars [38,39]. The combination of slag with ash had a particularly positive effect on strength parameters [39,48]. However, mortars with MSWI slag alone exhibited lower mechanical parameters after 2 and 28 days of curing compared to those containing ground blast-furnace slag.

In general, the composition of MSWI ash and slag depends on the type of MSW being incinerated and the parameters of the thermal conversion process. It is not surprising that these materials have attracted and continue to attract the interest of many researchers. However, there are far fewer reports on the management of other types of waste, including solid residues from the flue gas cleaning process in MSW incineration. This material was used in cement mortars by Lee et al. [49,50,51] after prior treatment. The waste was fused with fly ash and foundry sand [49], glass frit [50], or waste materials with significant SiO_2_ content [51], and then ground. Mortars synthesized with prepared ground slag sinter used as a cement substitute exhibited comparable or higher compressive strength compared to ordinary Portland cement mortars. The authors concluded that slag produced with foundry sand and glass frit, combined with fly ash and MSWI scrubber ash, provides a cost-effective and environmentally friendly substitute for cement in cement mortars. These findings are important, as recycling scrubber ash alone is not feasible, and melting it is highly challenging [49,50,51]. Thus, incorporating these wastes into cement mortar production may offer a viable solution for the closed-loop management of these materials. Another interesting approach is combining residues from municipal waste incinerators with residues from washing contaminated soil to produce concrete meeting C25/30 class parameters [52].

Generally, all the potential use of waste (by-products) as substitutes for natural materials deserves attention. Despite numerous scientific publications, specific proposals for effectively managing waste generated during the thermal processing of municipal solid waste remain lacking. Many studies, both within the conducted literature review and review articles [15,16,17], primarily concern bottom and fly ash, while only a few focus on other waste materials from this process or their applications in various combinations. The method of managing ash, slag, and other waste largely depends on their potential to release heavy metals into the environment, which is essential for assessing possible environmental hazards, as well as their physicochemical stability. Unfortunately, the high variability in the chemical composition of waste generated in the MSWI process hinders the development of standardized formulations suitable for use in building materials. Unfortunately, the high variability in the chemical composition and the presence of hazardous metal ions in waste from the MSWI process makes it difficult to develop a recipe that meets the standard requirements for building materials. However, the presence of hazardous metal ions does not disqualify these wastes, as shown in studies on the use of fly ash from biomass combustion [53,54] and waste from sewage sludge [55,56] in cement-based composite materials. The significant variability of waste composition makes it difficult to develop recipes for practical use. Therefore, statistical modeling is used to correct this variability, allowing the prediction of the technological properties for the final product in the most pessimistic scenario [57,58].

In this study, an attempt was made to explore the potential of using two types of hazardous waste (fly ash combined with solid waste from the flue gas cleaning process) for producing cement mortars. It is worth emphasizing that these materials were used without pretreatment, which typically increases process costs in industrial applications. Additionally, the use of waste from the gas purification process is noteworthy, as its safe and economical management remains highly problematic. In practice, such waste is neutralized through chemical stabilization and solidification and subsequently deposited in landfills or mine excavations, which is inconsistent with the principles of a circular economy.

## 2. Materials and Methods

### 2.1. Materials

**Figure 2:**
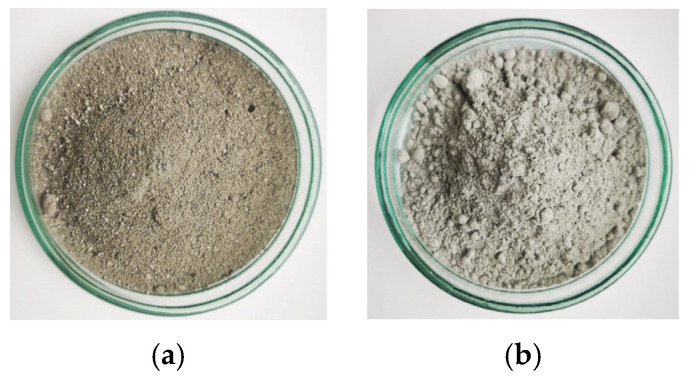
The MSWI-FA (**a**) and MSWI-SW (**b**) from the incineration of solid municipal waste.

The tested mortars were produced using Portland cement CEM I 42.5 R (Cemex; PN-EN 197-1 standard [59]); sand (with a particle size of 0–2 mm; standard PN-EN 196-1 [60]); water from an intake in Czestochowa (PN-EN 1008:2004 standard [61]); and waste from the incineration process of municipal solid waste. Two types of waste were used in the tests for modifying cement mortars: fly ash (MSWI-FA) and solid waste from gas treatment (MSWI-SW), derived from a grate-fired furnace installation (Figure 2) at a Polish municipal solid waste treatment plant. These wastes are by-products of the production process and are not utilized by the plant.

The MSWI-SW sludge was dried in a dryer at 105 °C for 24 h (POL-EKO type SLW 240-W STD) and then sieved. Materials with a grain size of <2 mm were used in the tests. The chemical composition of the MSWI waste, obtained using an XRF X-ray spectrometer (Thermo Fisher Scientific, Waltham, MA, USA), is presented in Table 2.

### 2.2. Research Methods

The control cement mortars and those containing a waste mixture used in this study were prepared in accordance with Polish standard PN 998 2:2016-12 [62]. The control mortar contained 1350 g of sand, 450 g of cement, and 225 g of water. A mixture of waste from incinerating solid municipal waste from a Polish plant—specifically, fly ash (MSWI-FA) and solid waste from the flue gas cleaning process (MSWI-SW)—was used as a sand substitute. Waste materials were used in proportions ranging from 0 to 20% by weight of cement, in line with the experimental plan described in a previous publication [63], which allows for comprehensive characterization of mortar properties across the full range of variability of controlled factors. The analysis methodology, actual values of controlled factors for coded values, and experimental plan without replication were consistent with those in previous research [63]. Statistical analysis was conducted using the STATISTICA v13.3 software and widely accepted analytical schemes [64]. The two controlled variables are fly ash (MSWI-FA) and solid waste (MSWI-SW). The observed parameters: consistency (C), flexural strength after 28 days (FS-28), compressive strength after 28 days (CS-28), water absorption (WA), mass loss after frost resistance tests (ML), and decreases in flexural (DFS) and compressive (DCS) strength following frost resistance tests.

The experimental design initially followed a Box–Wilson central composite design (CCD), modified by incorporating four additional far corner points at the axial levels. This resulted in five levels for the controlled factors. The design comprised 13 unique points, including 7 replicates at the center point to estimate random noise (pure error) caused by uncontrolled or minimally controlled factors. Employing statistical methods to optimize the composition of concrete mortars significantly reduces research costs, time, and labor while enhancing the reliability of the findings. This methodology has been validated in the design of steel–concrete structures [65] and studies on mortars containing metallurgical waste [63].

The properties of cement mortars, prepared in 4 × 4 × 16 cm samples, were assessed in accordance with relevant standards. All parameters were designated in compliance with the standards. Consistency was evaluated following PN-EN 1015-3 [66], while compressive and flexural strength were tested following PN-EN 1015-11:2020-04 [67] using an MMC-3742 testing machine (FPR; ToniTechnik 2030, Berlin, Germany). Mechanical strength tests were conducted on bars with dimensions of 40 × 40 × 160 mm. The bars were stored in molds for 24 h in an air-conditioned room at a temperature of 20 ± 1 °C and a relative humidity of at least 90%. After demolding, they were submerged in water at a temperature of 20 ± 1 °C until testing. Compressive and bending strength tests were carried out after 7 and 28 days of sample maturation. The bending strength test was performed on three bars, with the load increased at a rate of 50 ± 10 N/s until the bar fractured. Compressive strength tests were conducted on the halves of the bars. The compressive force was applied uniformly, increasing at a rate of 2400 ± 200 N/s. Water absorption and frost resistance (measured after 25 freeze–thaw cycles) were determined according to PN-85/B-04500 [68] using the Toropol K-010 climatic chamber (Toropol, Warsaw, Poland). Additionally, the study included a metal ion leaching analysis—standard PN-EN-12457-2:2006 [69] using Agilent MP-AES 4200 micro-wave-induced plasma atomic emission spectrometer.

## 3. Results

### 3.1. Summary of Results Used in Numerical Analysis

The by-products of the municipal solid waste (MSW) incineration process are becoming increasingly interesting to researchers in terms of their potential use in the construction sector, other than as ballast material in road or embankment construction. Of all the waste from the MSWI process, the most studied is bottom ash, which is generated in the largest amount and contains a lower concentration of hazardous substances than fly ash or solid waste from the flue gas cleaning process. However, considering the high content of CaO and SiO_2_ in these waste materials, research was carried out on their use as a substitute for sand in cement mortars. Using the MSWI-FA (19 01 13*) and MSWI-SW (19 01 07*) waste mixing plant in the amount of 20% each, we save about EUR 10 in sand purchase costs (which is 13% of the production costs of 1 m^3^) and EUR 8 in environmental fees when producing 1 m^3^ of mortar. In the first stage of the research, the mechanical properties of cement mortars synthesized using a mixture of both of these hazardous wastes were determined, and in the second stage, a test for leaching metal ions from the cement matrix was conducted. The tested parameters and the results that were obtained and submitted for numerical analysis are summarized in Table 3.

### 3.2. Analysis of Mortar Consistency

The consistency (C) of wet mortar, which binds construction products into a stable structure, is crucial for the durability of the structure. Consistency affects workability (the ease of spreading and shaping the mortar), adhesion (to bricks and aggregates), and strength (too little or too much water interferes with the proper binding process) [70].

**Figure 3:**
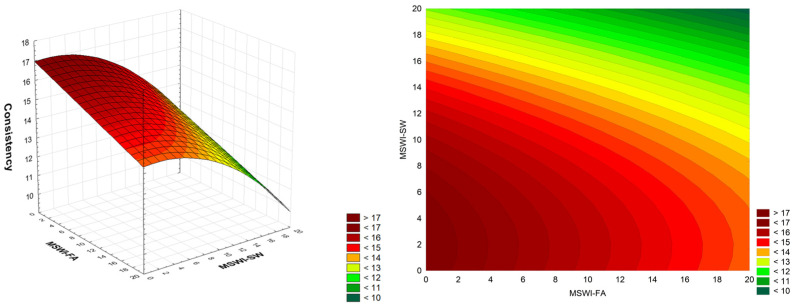
Dependence of the consistency of cement mortar on the amount of added MSWI-FA and MSWI-SW waste, in [%]; consistency [cm]; (3D and 2D graph).

The relationship between the consistency of cement mortars and the amount of added MSWI-FA and MSWI-SW waste is presented in Figure 3. As indicated by the analysis, an increase in the addition of MSWI-FA or MSWI-SW results in a decrease in the consistency of the produced cement mortars. All cement mortars modified with the addition of a mixture of ash and solid sludge from municipal waste incineration exhibit lower consistency than the control mortar. The obtained consistency of the control mortar series (PN-EN 1015-3) was 15.9 cm. Meanwhile, mortars containing 20% MWSI-FA measured 11.9 cm, and mortars containing 20% MWSI-SW measured 14.7 cm. Mortars containing a mixture of both wastes, with 20% of each, had a flow rate of 10.5 cm. Mortars containing the addition of waste MSWI-FA show a clear decrease in consistency, which can be explained by the small size of the added ash grains, with a regular, round shape. Such a mechanism was also observed in the work [18,37]. In addition, mortars modified with waste show a higher water demand than those made from natural aggregates.

The full quadratic RSM model for two factors (MSWI-FA, MSWI-SW, MSWI-FA × MSWI-FA, MSWI-SW×MSWI-SW, MSWI-FA × MSWI-SW) was adopted for the analysis. Variance analysis showed that the full model achieved statistical significance only for the linear components and the quadratic component of the MSWI-SW factor. After reducing the model using the backward stepwise regression method, the final model (MSWI-FA, MSWI-SW, MSWI-SW × MSWI-SW) was obtained, consisting of three statistically significant components. The model’s coefficient of determination was R^2^ = 0.85. The coefficients of the fitted model include a constant component (mean process response), linear components (MSWI-FA and MSWI-SW), and a quadratic component (MSWI-SW × MSWI-SW). Variability associated with the linear SW and FA terms is dominant:

C = 16.960 – 0.1318·MSWI-FA + 0.0509·MSWI-SW – 0.01384·MSWI- SW·MSWI-SW(1)

In its current reduced form, the model includes only statistically significant components, with residuals consistent with a normal distribution (Ryan–Joiner test, *p* > 0.1 *p* > 0.1) and a statistically insignificant lack-of-fit (*p* = 0.32).

A decrease in mortar consistency was also observed by Thomas and Ślosarczyk [38] when sand was replaced with 20% or 50% MSWI slag. Mortars containing 50% slag were characterized by lower plasticity due to the greater porosity, lower density, and higher water demand of the slag as an aggregate, compared to mortars containing 20% of this additive, regardless of the type of cement used (CEM 42.5 or CEM 52.5). Similar results were reported by Cheng et al. [71], who used ground MSWI bottom ash with a grain size of <4.75 mm as a sand substitute in amounts ranging from 10% to 40%. Mortar consistency decreased from 131 mm (control mortars) to 101 mm (for mortars with 40% substitution).

### 3.3. Flexural and Compressive Strength Cement Mortar Analysis After 28 Days

**Figure 4:**
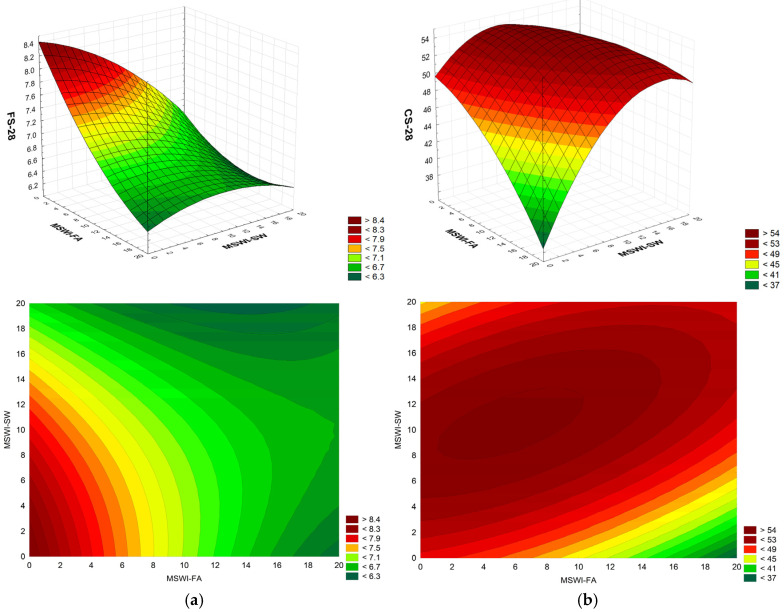
Dependence of the flexural (**a**) and compressive (**b**) strength of mortars after 28 days on the amount of added MSWI-FA and MSWI-SW in [%}; FS-28 and CS-28 in [MPa]; (3D and 2D graph).

The compressive and flexural strength of cement mortars is crucial, as it determines their ability to transfer loads and resist various operating conditions, ensuring the stability and reliability of the structure throughout its service life. These parameters are, therefore, most often tested in relation to newly synthesized materials. The changes in the flexural and compressive strength of the modified mortars in relation to the amount of replaced sand are shown in Figure 4.

The full quadratic RSM model for two factors (MSWI-FA, MSWI-SW, MSWI-FA × MSWI-FA, MSWI-SW × MSWI-SW, MSWi-FA × MSWI-SW) was adopted for the analysis. The analysis of variance showed that the full model met statistical significance for all model components. The model’s determination coefficient was R^2^ = 0.93. Variability associated with the linear components of MSWI-FA and MSWI-SW dominates:

FS-28 = 8.410 − 0.1612·MSWI-FA − 0.0315·MSWI-SW + 0.00287·MSWI-FA·MSWI-FA − 0.00253·MSWI-SW·MSWI-SW + 0.004151·MSWI-FA·MSWI-SW(2)

In its current form, the model has only statistically significant components and model residuals consistent with the normal distribution (Ryan–Joiner test, *p* > 0.1), with a statistically insignificant lack of fit (lack-of-fit test, *p* = 0.46).

The compressive strength was also analyzed. The full quadratic RSM model for two factors (MSWI-FA, MSWI-SW, MSWI-FA × MSWI-FA, MSWI-SW × MSWI-SW, MSWI-FA × MSWI-SW) was adopted for the analysis. The analysis of variance showed that the full model was statistically significant for all components. The model’s coefficient of determination was R^2^ = 0.91. The variability associated with the quadratic MSWI-SW component and the synergistic interaction MSWI-SW × MSWI-FA dominated:

CS-28 = 49.58 − 0.209·MSWI-FA + 1.016·MSWI-SW − 0.02237·MSWI-FA·MSWI-FA − 0.06047·MSWI-SW·MSWI-SW + 0.04424·MSWI-FA·MSWI-SW(3)

In its current form, the model includes only statistically significant components, with residuals consistent with a normal distribution (Ryan–Joiner test, *p* > 0.1) and no statistically significant lack of fit (lack-of-fit test, *p* = 0.26).

After 28 days of hardening, the flexural strength of cement mortars containing 20% fly ash and 20% solid sediment is lower by 23.2% and 24.9%, respectively, compared to the control mortars. On the other hand, the flexural strength of cement mortars containing a mixture of both wastes (20% each) is lower by 25.9% compared to the control samples. Similarly, after 28 days of hardening, the compressive strength of cement mortars modified with the addition of both wastes also decreases. The compressive strength of cement mortars containing 20% fly ash and 20% solid sediment is lower by 27.7% and 5.3%, respectively, compared to the control mortars. However, the compressive strength of cement mortars containing a mixture of both wastes (20% each) is only 1.3% lower than the control mortars.

A special feature observed during the tests is the ambiguous response in the mechanical strength (compressive strength, flexural strength) after the frost resistance tests. In the case of flexural strength, both decreases up to 22% and increases up to 34% were observed. Similarly, in the case of compressive strength, decreases up to 10% and increases up to 10% were observed.

The behavior of both values in the case of replication tests (MSWI-FA = 10%, MSWI-SW = 10%, number of replications 7) is especially interesting. Here, decreases of up to −13% and increases of up to 25% were observed for flexural strength. Similarly, decreases of up to −8% and increases of up to 5% were observed for compressive strength. This indicates that the percentage share of both components alone is insufficient to describe the behavior unambiguously after frost resistance tests. It is necessary to identify additional material properties or process factors in the future to allow for a narrower prediction of this behavior.

The synthesized cement mortars with fly ash (MSWI-FA) and solid waste (MSWI-SW) mixtures exhibit lower strength parameters compared to the mortars synthesized by Al-Rawas et al. [45], using fly ash and button ash mixtures. The mortar synthesized with the use of ash mixtures (particle size 0.075 ÷ 4.75 mm) as a substitute for sand (at levels of 0–40%) and cement (at levels of 0–30%) demonstrated higher compressive strength than the control mortars. The maximum compressive strength (36.4 N/mm^2^) after 28 days was achieved by composites containing 20% ash. Pietrzak [72] reports that mortars containing 30% fly ash from the MSWI process show a decrease in compressive strength by 8% and bending strength by 21.5% compared to control mortars.

Tian et al. [43] also observed the positive effects of the ash mixture on mortar and concrete properties. Replacing up to 100% of the natural aggregate with coarse fractions (9.5–25 mm) and medium (2–9.5 mm) fractions of fly ash and bottom ash mixtures yielded concrete with compressive strength exceeding 28 MPa after 28 days of curing. Similar results were obtained when using a fine ash fraction (<2 mm) as a sand substitute at 10% by weight [43]. Additionally, fly ash from MSWI was combined with municipal sludge (30%, 60%, and 90%), resulting in mortars with compressive strengths ranging from 29.5 to 44.1 MPa after 28 days of curing [48]. Additionally, MSWI-FA has been used in slag–cement mortars [34] after prior melting in an electric furnace at 1450 °C for one hour and grinding. Adding MSWI-FA to slag–cement mortar as a partial Portland cement substitute (10–40%) along with CaCO_3_ (10–40% by weight) results in composites with compressive strength after 14 and 28 days similar to reference mortars and with higher long-term strength (after 90 days) due to the pozzolanic reaction.

Fly ash MSWI-FA has also been used in concrete. Bertolini et al. [18] found that part of the Portland cement could be replaced by MSWI fly ash after pretreatment (rinsing with water to reduce chloride content). In another study, Poranek et al. [37] used MSWI-FA (5.8% of binder mass) combined with clinoptilolite zeolite (to immobilize metal ions) in cement mortars. After 28 days, non-aerated composites containing MSWI-FA exhibited lower compressive strength (49.5 MPa) than control samples (53.2 MPa). In aerated mortars, the cement composite containing MSWI-FA also showed reduced compressive strength (47.2 MPa) compared to the control sample (52.8 MPa), likely due to the ash’s role as a filler rather than a binding material. However, an increase in compressive strength in concretes containing MSWI waste can be achieved by introducing 0.1% polypropylene fibers [41].

Slag from MSWI (MSWI slag) has also been used as a substitute for natural raw materials in concrete [46,47] and mortar [38,39]. Slag was added to concrete in combination with ash as well [51]. Thomas [38] showed that using MSWI slag as a substitute for natural aggregate in amounts up to 50% by volume decreased workability and increased water absorption in cement mortars, regardless of the cement type. Mortars made with CEM 42.5 cement exhibited compressive strength after 28 days, comparable to control samples, while those with CEM 52.5 cement showed significant strength differences, with the highest strength observed in composites containing 50% slag. Czop et al. [6] reported that mortars with MSWI slag have lower mechanical properties after 2 and 28 days of curing than mortars with ground blast-furnace slag. However, these mortars showed a 3% higher water absorption rate compared to those with ground granulated blast-furnace slag, and their cement paste setting time was extended by 125 min. Mortars containing 30% MSWI slag demonstrated lower mechanical strength after 28 days of curing than both the control mortars and those containing blast-furnace slag, with compressive and flexural strengths of 32.0 MPa and 4.0 MPa, respectively (compared to 45 MPa and 6 MPa for the control samples). Chemical testing indicated that using MSWI slag in mortars would not pose environmental hazards, and this material exhibits many physical and chemical properties similar to crushed, granulated blast-furnace slag commonly used in mortars.

Meanwhile, Ferraris et al. [21] showed that vitrified bottom ash (VBA), heated to 1450 °C without a vitrifying agent, is unsuitable as a sand replacement in concrete. However, when up to 20% by weight of VBA with a vitrifying agent is used as a cement substitute and up to 75% by volume as a natural aggregate replacement, the resulting concrete exhibits mechanical properties comparable to control concrete. Furthermore, no alkali-silica reactions or other harmful effects were observed in concrete samples containing VBA, even after two years. Similarly, studies by Saikia et al. [73] indicate that pretreating waste material enhances the mechanical properties of cement mortars. When a mixture of MSWI-BA and boiler and fly ash from a fluidized bed incinerator (BFA) (grain size 0.1 ÷ 2 mm) undergoes chemical treatment (0.25 M Na_2_CO_3_ solution) and combined chemical and thermal treatment at approximately 675 °C, mortars incorporating this waste as a sand substitute show higher compressive strength than control samples. Pera et al. [23] further demonstrated that introducing up to 50% pretreated bottom ash (4–20 mm) as a gravel substitute yields concrete with adequate strength (25 MPa after 28 days). Pre-milled wet bottom ash also shows potential as a mineral additive for concrete production [18]. In contrast, untreated bottom ash (without vitrification) can lead to concrete degradation due to reactions between aluminum and cement paste, resulting in the formation of aluminum hydroxide, aluminates, and hydrogen gas [22].

In general, the composition of MSWI ash and slag depends on the nature of the municipal waste being incinerated and the parameters of the thermal conversion process. Thus, it is not surprising that these materials have attracted, and continue to attract, the interest of many researchers. However, there are significantly fewer reports on the management of other waste types, including solid residues from the flue gas cleaning process in municipal solid waste incineration. Lee et al. [50,51] used specially prepared ground slag (<38 μm) obtained by melting a mixture of fly ash and MSWI scrubber ash (in a 1:3 ratio) with waste fly ash from foundry sand (to achieve a CaO/SiO_2_ alkalinity of 1.1) to produce cement mortars. Cement mortars containing 10–40% of the prepared slag powder (used as a cement substitute) exhibited higher compressive strength than the control samples after 28 and 90 days (by approximately 24–48%) due to the pozzolanic reaction of the added slag. Additionally, fly ash and ash from the MSWI scrubber (in a 1:3 ratio) were sintered with glass frit to obtain slag, which, after grinding (<38 μm), was used to produce cement mortars [49]. The compressive strength of mortars containing the prepared slag as a cement substitute (20–40%) was comparable to that of ordinary Portland cement mortars after 28 days, while the strength after 60 and 90 days was higher by 3–11%. Similar effects were observed when using slag produced from MSWI fly ash, scrubber ash, and waste materials with a significant SiO_2_ content [49,50,51]. The compressive strength of mortars containing this substitute after 28 days of curing was nearly identical, and after 60 and 90 days, it was higher (by 2 ÷ 11%) compared to the control mortars.

Moreover, the produced mortars were a good matrix for immobilizing hazardous metal ions. The fact that cement mortars modified with mix waste (MSWI-FA and MSWI-SW) meet the requirements for mechanical properties justifies further research on these synthesized materials in terms of their environmental safety.

### 3.4. Water Absorption Analysis of Cement Mortars Containing MSWI-FA and MSWI-SW

**Figure 5:**
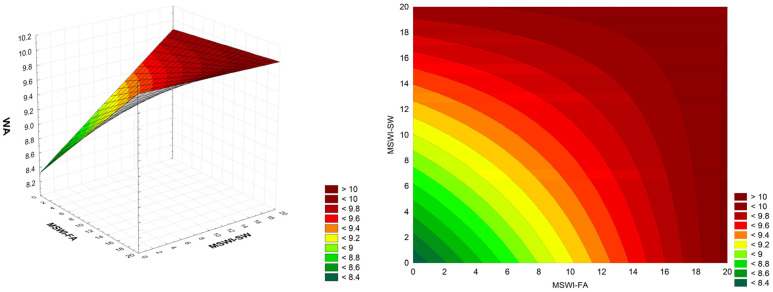
Dependence of water absorption on the amount of MSWI-FA and MSWI-SW in the cement mortar, MSWI-FA and MSWI-SW in [%], WA in [%]; (3D and 2D graph).

The next parameter analyzed was water absorption in mortars, which affects the material’s mechanical properties, durability, and resistance to environmental impacts. Water penetrating the pores of cement mortar can freeze and cause microcracks, reducing the structure’s durability. The water absorption of the tested mortar samples, depending on the proportion of the added waste mixture, is shown in Figure 5.

The quadratic components in the initial full quadratic model were statistically insignificant. After reduction, the model has linear components and an antagonistic two-way interaction. ANOVA analysis revealed that the linear components of MSWI-FA and MSWI-SW dominate (F-value 31.5 and 21.2, respectively), and the interaction effect is slightly weaker (F-value 14.8), while the model accuracy is satisfactory (lack-of-fit *p*-value = 0.31). The model residuals are consistent with the normal distribution (Ryan–Joiner test *p*-value = 0.09) and the coefficient of determination R^2^ = 0.82.

WA = 8.319 + 0.0860·MSWI-FA + 0.0777·MSWI-SW − 0.00399·MSWI- FA·MSWI-SW(4)

The water absorption of the control mortars was 8.07% and increased slightly with the amount of waste added. The absorption of mortars with 20% MSWI-FA and 20% MSWI-SW was 9.79% and 9.98%, respectively. In contrast, the absorption of mortars containing a mixture of both wastes, at 20% each, was 10.15%. A slight increase in water absorption (0.3 ÷ 1.6%) was reported by Thomas and Ślosarczyk [38] in the production of cement mortars (CEM 42.5 and CEM 52.5) with 20% slag from the MSWI process. Mortars based on CEM 52.5 exhibited lower water absorption than those based on CEM 42.5. However, the water absorption of CEM 42.5 mortars increased after the frost resistance test (by 0.8 and 1.6 pp, respectively).

### 3.5. Analysis of the Mechanical Strength of Cement Mortars After Frost Resistance Tests

The frost resistance testing of cement mortars allows for the assessment of their suitability in variable climatic conditions where freeze–thaw cycles occur regularly. These processes directly affect the durability and safety of building structures. Therefore, the decrease in flexural and compressive strength of mortars modified with additions from the MSWI process was assessed.

**Figure 6:**
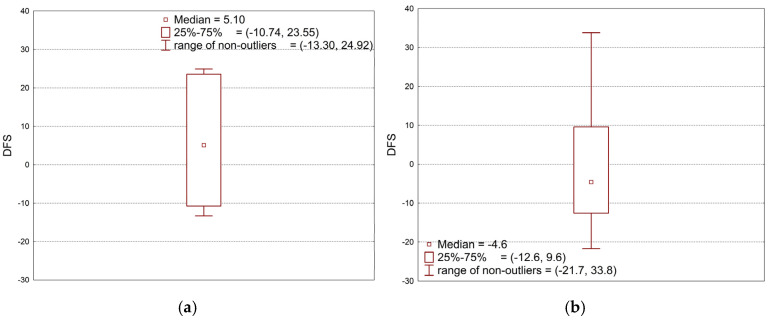
Variability of DFS observed data caused by non-controlled factors (**a**) compared to total variability of data caused by controlled factors (**b**).

Data analysis was performed using the full quadratic model. The R^2^ coefficient of determination was obtained at a value of only 0.45. In-depth analysis of the replicated data (MSWI-FA = 10%, MSWI-SW = 10%, seven replications) showed a very large scatter of results around the zero value (Figure 6a), despite the fact that this concerns the established values of the controlled variables (mean 5.86, standard deviation 16.00). Comparison of the variability of the replicated data with the total variability of the collected data (Figure 6b; mean −0.40, standard deviation 15.75) showed that the variability from controlled factors (MSWI-FA, MSWI-SW) is too small in relation to the variability from uncontrolled factors to create a reliable prognostic model. The observed characteristic feature is the occurrence, with the same settings of controlled factors, of both strengthening and weakening of torsional strength after frost resistance tests in a wide range from −13% to +25%.

**Figure 7:**
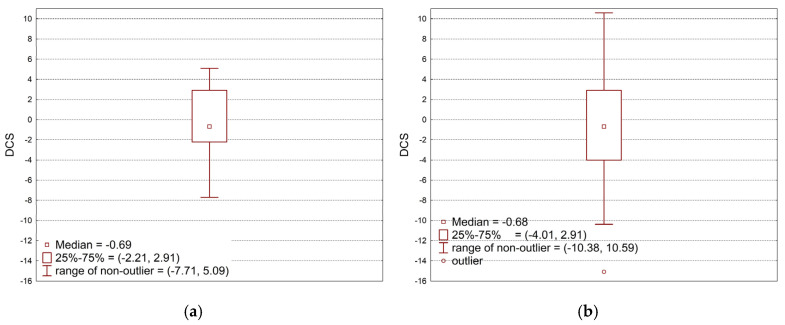
Variability of DCS observed data caused by non-controlled factors (**a**) compared to total variability of data caused by controlled factors (**b**).

Also, for compressive strength, data analysis was performed using the full quadratic model. The R^2^ coefficient of determination was obtained at a value of only 0.51. In-depth analysis of the replicated data (MSWI-FA = 10%, MSWI-SW = 10%, seven replications) showed a very large scatter of results around the zero value (Figure 7a), despite the fact that this concerns the established values of the controlled variables (mean −0.46, standard deviation 4.16). Comparison of the variability of the replicated data with the total variability of the collected data (Figure 7b; mean −0.69, standard deviation 6.28) showed that the variability from controlled factors (MSWI-FA, MSWI-SW) is too small in relation to the variability from uncontrolled factors to create a reliable prognostic model. The observed characteristic feature is the occurrence, with the same settings of controlled factors, of both strengthening and weakening of compression strength after frost resistance tests in a wide range from −8% to +5%.

The data obtained show that all cement mortars modified with the addition of the MSWI-FA and MSWI-SW waste mixture exhibit a smaller increase/decrease in bending strength compared to the control mortar after frost resistance tests. The bending strength of the control samples of cement mortars decreased by 21.5% after 25 freeze–thaw cycles, while mortars containing 20% MSWI-FA or 20% MSWI-SW exhibited reductions of 13.8% and 8.7%, respectively. Mortars containing a mixture of both wastes (20% each) showed a decrease in bending strength of only 5.8%. In the case of compressive strength, a much smaller decrease was observed for cement mortars modified with these wastes. For mortars containing a mixture of 20% MSWI-FA and 20% MSWI-SW, the decrease was 1.68%, while for mortars containing MSWI-FA and MSWI-SW individually, the reductions were 10.4% and 0.7%, respectively.

The obtained results are difficult to compare with literature reports, as the frost resistance of cement composites containing MSWI waste has been rarely studied. Thomas and Ślosarczyk [38] observed a smaller decrease in compressive strength after 150 freeze–thaw cycles in mortars containing 20% MSWI slag (regardless of the type of cement used) compared to control mortars. This decrease was smaller in mortars made with CEM I 52.5 cement. The largest percentage decrease (26.1%) was noted for control samples made with CEM I 52.5 cement, while all samples made with CEM I 42.5 cement exhibited a strength reduction of 12%. The smallest decrease in strength (0.4%) was observed in mortars based on CEM I 42.5, which contained 50% slag as aggregate. The research conducted by Keppert et al. [74] showed that there was no deterioration in frost resistance of concretes produced with 10% slag as aggregate MSWI after both 75 and 125 freeze–thaw cycles. The porous structure of the slag acted as an aerating admixture, increasing the space content for the freezing water.

### 3.6. Mass Loss (ML) Analysis of Cement Mortar with MSWI

**Figure 8:**
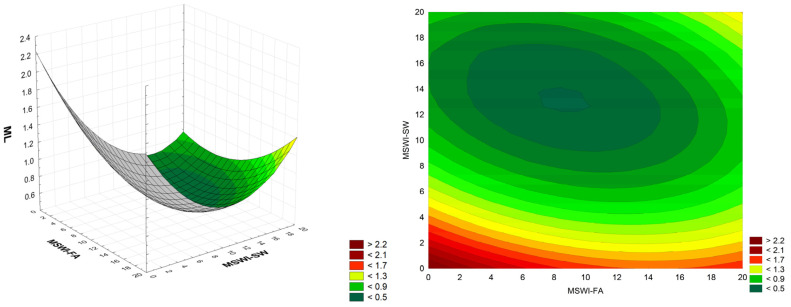
Dependence of the mass loss of cement mortars after frost resistance tests on the amount of added MSWI-FA and MSWI-SW; ML in [%], MSWI-FA and MSWI-SW in [%]; (3D and 2D graph).

The next parameter analyzed was the mass loss of modified cement mortars after the freeze–thaw test. The study of mortar mass loss provides valuable information about their durability and susceptibility to degradation under variable climatic conditions. The analysis revealed that the tested cement mortars modified with the addition of MSWI-FA and MSWI-SW waste exhibited small fluctuations in mass loss after frost resistance tests (Figure 8).

The linear MSWI-FA component in the initial full quadratic model was found to be statistically insignificant. ANOVA analysis for the reduced model revealed that two components, linear and quadratic MSWI-SW, dominate (F-value 31.9 and 31.2, respectively), while quadratic MSWI-FA and the two-way synergistic interaction (F-value 8.6 and 6.3, respectively) have a much smaller influence, while the model accuracy is still acceptable (lack-of-fit *p*-value = 0.06). The model residuals are consistent with the normal distribution (Ryan–Joiner test *p*-value > 0.1) and the coefficient of determination R^2^ = 0.88.

ML = 0.06047·MSWI-SW·MSWI-SW + 0.04424·MSWI-FA·MSWI-SW(5)

Studies by Thomas and Ślosarczyk [38] similarly reported minimal changes in mortars modified with MSWI slag. Some samples exhibited a slight mass gain (ranging from 1.19% to 1.93%) after the freeze–thaw test, while others showed a slight mass loss (ranging from (−1.12)% to (−2.45)%). The maximum percentage mass gain occurred in mortars containing 20% slag as aggregate based on CEM 42.5, while the maximum percentage mass loss was observed in mortars based on CEM 52.5 with 50% slag as aggregate. At the same time, Al-Rawas et al. [45] reported that the mortar samples with ash as a sand substitute showed lower mass loss values than the control mortars.

### 3.7. Metal Ion Leaching Test from Mortars Containing MSWI

The concentration of leached heavy metals is a key indicator for assessing the safety of building materials synthesized from waste. This assessment is particularly important when waste containing toxic metal ions is used for synthesis. Consequently, many studies on the utilization of MSWI waste evaluate products from this perspective. It should be noted, however, that the authors of the studies refer their results to various legal regulations in force in individual countries (Table 4), which may distort the perspective of their comparison and interpretation. Additionally, these criteria may sometimes refer to waste intended for landfilling (e.g., in inert or hazardous waste landfills) or, in other cases, to permissible levels in discharged waters.

In this study, a leaching test was performed to evaluate metal ion release from cement mortars synthesized with a maximum (20%) content of added MSWI-FA or MSWI-SW waste, as well as a combined maximum content of both wastes (total 40%). This approach assumed that the highest concentration of metal ions in the eluate would be observed under such conditions. The tests were conducted on cement mortar samples after 28 days of hardening in accordance with the PN-EN 12457-2:2006 standard. According to the standard, 95% of the sample mass should consist of grains with a size of less than 4 mm. For this purpose, concrete samples were crushed on a jaw crusher and sieved through a sieve with a mesh size of 4 mm. From the sample prepared in this way, an analytical sample (S) weighing 0.090 ± 0.005 kg was taken and placed in a vessel (bottle), and then distilled water (L) with an electrical conductivity of 0.45 mS/m in the amount of 0.9 dm^3^ was added to it. According to the standard, the ratio of the eluting liquid (L) to the solid phase (S) should be 10 dm^3^ to 1 kg. The closed vessel was placed in a mixing device (roller table) for 24 h ± 0.5 h. Then the vessel was left for 15 ± 5 min to allow the suspension to settle. The effluent was filtered through a membrane filter with a pore diameter of 0.045 µm. After the leaching test, the pH of the eluate from the control mortars was 12.19, while for mortars modified with waste, it ranged between 12.25 and 12.29. The results of the metal ion concentrations in the eluate (Table 5) did not exceed the permissible values for introducing substances particularly harmful to the environment into sewage or soil, as defined by the Regulation of the Minister of Maritime Economy and Inland Navigation of 12 July 2019 [75], with the exception of barium ions.

The immobilization level of metal ions in the cement mortar matrix was high, ensuring that even damaged cement mortars containing the maximum amount of MSWI-FA and MSWI-SW waste in contact with water would not pose an environmental threat. High immobilization of heavy metal ions in monolithic cement mortars with the addition of waste from the purification of waste gases from municipal waste incinerators is also reported by Czop et al. [13]. The degree of immobilization of the tested metals (Zn, Ni, Co, Cd, Fe, Mn) in the C–S–H phase reached 99.9%. A high level of immobilization of chloride and sulfate ions was also observed, with their concentrations, like those of metal ions, remaining below the permissible limits for inert waste landfills (the soil standard allows 7 mg/kg barium ions).

**Table 4:** Legal regulations used in assessing the impact of MSWI waste on the environment in publications in the field of cement composites.

Ref.	Legal Act	Units	Zn	Ni	Cd	Pb	Cr	Ba	As	Se	Cu
[25]	EPA [76]	mg/dm^3^	-	-	-	5.0	5.0	100	5.0	1.0	-
[25]	National THA * [77]	mg/kg	-	1600	37	400	300	-	3.9	390	-
[50]	TCLP [78]	mg/dm^3^	-	-	1	5	5		-	-	15
[6]	EU ** [79]	mg/kg	4	0.4	0.04	0.5	0.5	20	-	-	2
[6]	EU *** [79]	mg/kg	50	10	1	10	10	1000			50
[47]	National [80]	mg/dm^3^	50	-	0.3	3.0	0.3	-	-	-	50
[81]	National [82]	μg/dm^3^	1000	20	5	10	50				1000
[77]	National [83]		2.8			1.3	0.5	-	0.8	0.1	0.5
[13]	National PL ** [84]	mg/kg	2.0	0.2	0.03	0.2	0.2	7.0	-	-	0.9

* Soil Quality Standards ** Landfills for Inert Waste *** Non-Hazardous Waste.

Czop and Łaźniewska-Piekarczyk [6] also reported high immobilization of metal ions in cement mortars containing 30% MSWI slag. The heavy metal content in MSWI slag was high (ranging from 1621.0 mg/kg for Zn to 0.24 mg/kg for Hg) and followed the order Cu > Zn > Pb > Cr > Ni > V > As > Cd > Tl > Hg. However, the concentrations of metals in the eluate from cement mortars hardened for 28 days containing this waste did not exceed the permissible values for Zn, Cd, and Ni, while the remaining metals were below the detection threshold (Ba, Cu, Cr, Co, Fe, Mn). The exception was lead (Pb), with a concentration (0.1 mg/kg) slightly exceeding permissible levels for inert waste landfills. The authors suggested that MSWI slag could be used in construction practice, as it meets most standard requirements as a substitute for cementitious material. Also, Lee et al. [34] confirmed a high level of immobilization of metal ions (Ni, Zn, Cd, Pb, Cr, and Cu) in mortars containing slag and fly ash. Metal concentrations in the eluate were below permissible limits. Conversely, Poranek et al. [37] observed high leachability of Ba ions (596 mg/dm^3^), Na (14,820 mg/dm^3^), K (1129 mg/dm^3^), and Ca (10,410 mg/dm^3^) from MSWI-FA waste. Phutthimethakul and Supakata [25] found that metal ion levels (Ba, As, Co, Cd, Fe, Cr, Mn, Cu, Se, Zn, Ni, Pb) in bottom ash eluate were within legal limits, concluding that additional leachability tests were unnecessary. Also, Saikia et al. [73] demonstrated that although the concentrations of metals (Cr, Cu, Mo, Ba, Zn, Pb) in eluates from cement mortar with bottom ash after 64 days of hardening were higher than those in the control sample, they did not exceed permissible values. Some authors propose preliminary waste processing, such as calcination and vitrification of MSWI-FA, to enhance safety [11,85]. It has been shown that the calcination and vitrification process of MSWI-FA reduces Cd and Pb ion concentrations by 95%, Cu ions by 85%, and Zn ions by 50% [11]. Additionally, the heat treatment process can eliminate up to 82% of Zn ions [85].

Therefore, based on the results of this study and literature data, it can be stated that the permissible level of pollutants introduced into water [75] in the case of barium ions were exceeded, but they do not exceed the required levels for waste designated for landfill [84]. Therefore, in the future, it is necessary to try to subject waste from waste gas treatment plants from the MSWI process to thermal or chemical treatment before introducing them into mortars or concretes.

**Table 5:** The ion leaching from cement mortar doped with the addition of 20% MSWI-FA and/or MSWI-SW waste.

Sample No	Waste	Units	Zn	Ni	Cd	Pb	Cr	Ba	Cu
Legal act [75] *	–	mg/dm^3^	2.0	0.5	0.4	0.5	0.5	2.0	0.5
Legal act [84] **	–	mg/dm^3^	2.0	0.2	0.03	0.2	0.2	7.0	0.9
No 1	Control	mg/dm^3^	0.245	<0.01	<0.01	0.292	<0.01	3.248	0.025
No 3	MSWI-SW	mg/dm^3^	0.110	<0.01	<0.01	0.241	<0.01	3.726	<0.01
No 5	MSWI-SW + MSWI-FA	mg/dm^3^	0.112	<0.01	<0.01	0.221	<0.01	3.682	<0.01
No 7	MSWI-FA	mg/dm^3^	0.072	<0.01	<0.01	0.216	<0.01	2.984	<0.01

* Into water or ground ** Landfills for Inert Waste.

## 4. Conclusions

The systematic increase in the amount of waste generated from the thermal processing of municipal solid waste necessitates the search for effective management strategies. These materials, given their high SiO_2_ and CaO content, can serve as substitutes for natural aggregates, aligning with the principles of the circular economy. Analysis of the obtained results indicates the following:

(1) Using a mixture of fly ash (MSWI-FA) and solid waste from the purification of flue gases (MSWI-SW) generated during the incineration of municipal waste, as a substitute for aggregate, enables the production of cement mortars with good strength parameters.
(2) The obtained models predicting compressive strength and flexural strength are of good quality, as the coefficient of determination of the models was 0.91 and 0.93, respectively, while the lack of fit had a significance level *p* of 0.26 and 0.46, respectively.
(3) The mortars containing a mixture of MSWI-FA and MSWI-SW (at a 20% replacement rate each) exhibit only a 1.3% reduction in compressive strength and a 25.8% reduction in bending strength.
(4) The flexural and compressive strength, after the frost resistance tests, showed ambiguous behavior, showing both increases and decreases, indicating that the percentage of waste components alone is an insufficient set of factors for predicting these strength properties.

The results also indicated that, after the leaching process, the concentration levels of the tested metal ions (Zn, Pb, Cr, Ni, Cd, Cu), except for barium, do not exceed the pollutant levels permitted by law for release into water and soil. Therefore, before the practical use of the MSWI-FA and MSWI-SW waste mixture in composites with a cement matrix, such as mortars or concretes, it should undergo prior thermal or chemical treatment. This research direction is worth considering, as the use of a mixture of these wastes, at 20% each, offers not only ecological but also practical benefits. Replacing sand with this waste in 1 m^3^ of mortar can save approximately EUR 18.0 in sand purchase costs and environmental fees. Therefore, the next aspect of our research will be to determine the impact of using selected polymer compounds in combination with the tested waste, enabling the immobilization of barium ions within the cement matrix. The primary limitation of this solution lies in the high cost of polymers, which will increase the production costs of mortars. However, the environmental benefits of effectively managing the steadily growing amount of waste significantly outweigh these costs.

The issue presented in the article is central to the development of a closed-loop economy. One can see the convergence and consistency of the fundamental objective of a closed-loop economy in terms of reducing waste and minimizing its impact on the environment, which is also in line with the concept of sustainable development. On the other hand, from the perspective of effective municipal waste management, a holistic view of businesses’ widespread transition to a closed-loop economy is relevant. The application of innovation by companies at the stage of sustainable product design and the creation and implementation of closed-loop solutions for the entire product life cycle seems to be of particular importance. It should be pointed out that actions taken by companies to minimize waste can bring tangible benefits from the perspective of their profitability and the municipal waste management system, which is in line with the closed-loop economy strategy. However, a key issue from a municipal waste management perspective remains the awareness of businesses in terms of investing in circular solutions that create a business model focusing on minimizing raw material consumption and waste generation. From a societal perspective, now and in the future, there is a need to educate and point out the economic benefits of the circular economy. It should be pointed out that circular practices can generate economic value and new business opportunities.

## Figures and Tables

**Table 1:** Waste generated in the process of thermal treatment of municipal waste.

Code		Used **
19 01	Wastes from Incineration or Pyrolysis of Waste [10]	
19 01 02	Ferrous materials removed from the bottom ash	
19 01 05*	Filter cake from gas treatment	
19 01 06*	Aqueous liquid waste from gas treatment and other aqueous liquid waste	
19 01 07*	Solid waste from gas treatment	X
19 01 10*	Spent activated carbon from flue gas treatment	
19 01 11*	Bottom ash and slag containing dangerous substances	
19 01 12	Bottom ash and slag other than those mentioned in 19 01 11	
19 01 13*	Fly ash containing dangerous substances	X
19 01 14	Fly ash other than those mentioned in 19 01 13	
19 01 15*	Boiler dust containing dangerous substances	
19 01 16	Boiler dust other than those mentioned in 19 01 15	
19 01 17*	Pyrolysis waste containing dangerous substances	
19 01 18	Pyrolysis waste other than those mentioned in 19 01 17	
19 01 99	Wastes not otherwise specified	

* hazardous waste. ** used for our research.

**Table 2:** Chemical composition of investigated MSWI-FA and MSWI-SW waste [%].

**Waste**	**SiO_2_**	**CaO**	**MgO**	**Al_2_O_3_**	**F_2_O_3_**	**Na_2_O**	**K_2_O**	**P_2_O_5_**	**S**	**Cl**
MSWI-SW 19 01 07*	4.85	43.97	2.95	7.31	0.72	4.11	1.05	0.57	4.24	8.21
MSWI-FA 19 01 13*	17.22	37.22	4.31	9.62	2.91	9.02	1.45	1.13	3.14	4.51
**Waste**	**Mn**	**Zn**	**Ti**	**Pb**	**Sn**	**Cu**	**Cr**	**Ba**	**Sr**	**Cd**
MSWI-SW 19 01 07*	0.04	1.08	0.79	0.11	0.04	0.04	0.02	0.11	0.03	0.01
MSWI-FA 19 01 13*	0.09	0.83	1.31	0.93	0.03	0.05	0.01	0.14	0.05	0.02

* hazardous waste.

**Table 3:** Measurement results for the assumed experimental plan: consistency (C), flexural (FS-28) and compressive (CS-28) strength after 28 days, water absorption (WA), mass loss after frost resistance tests (ML) and decreased flexural strength (DFS) and compression strength (DCS) after frost resistance tests of mortars (more information can be found in the Supplementary Material).

No.	MSWI-FA	MSWI-SW	C	FS-28	CS-28	WA	ML	DFS	DCS
	[%]	[%]	[cm]	[MPa]	[MPa]	[%]	[%]	[%]	[%]
1	0.00	0.00	16.1	8.57	50.42	8.07	2.39	−21.5	−4.0
2	0.00	10.00	15.2	7.75	50.67	9.29	0.90	33.8	−3.5
3	0.00	20.00	11.9	6.58	47.75	9.79	1.08	−13.8	−10.4
4	10.00	20.00	11.0	6.29	49.79	9.93	0.72	−8.7	3.2
5	20.00	20.00	10.3	6.36	49.78	10.15	1.21	−5.8	1.6
6	20.00	10.00	12.4	6.52	50.97	9.89	1.07	1.9	10.2
7	20.00	0.00	14.6	6.44	36.46	9.98	1.32	−6.9	−0.7
8	10.00	0.00	15.1	6.90	44.23	9.78	2.01	−21.7	−15.1
9	10.00	10.00	14.7	7.22	54.30	9.50	0.43	−4.6	−7.7
9	10.00	10.00	14.5	6.91	52.65	9.60	0.52	−10.7	−2.1
9	10.00	10.00	13.8	7.15	55.91	9.75	0.79	23.5	−2.2
9	10.00	10.00	14.4	6.75	51.96	9.28	0.55	−13.3	1.4
9	10.00	10.00	15.0	6.81	54.97	9.71	0.40	5.1	−0.7
9	10.00	10.00	15.9	6.91	53.98	9.24	0.52	16.1	2.9
9	10.00	10.00	15.7	6.86	55.15	9.39	0.49	24.9	5.1
10	2.93	2.93	18.3	7.77	52.88	8.70	1.24	−12.6	1.2
11	2.93	17.07	14.4	7.21	49.40	9.83	0.70	−3.1	1.2
12	17.07	17.07	11.1	6.39	51.64	9.96	0.99	9.6	10.6
13	17.07	2.93	15.1	6.62	42.88	9.72	1.23	0.3	−4.2

## Data Availability

The original contributions presented in this study are included in the article. Further inquiries can be directed to the corresponding author.

## Supplementary Materials

The following supporting information can be downloaded at: https://www.mdpi.com/article/10.3390/ma18030481/s1. The original raw data analyzed in the article are included in supplementary dataset.
